# Physical, Chemical and Sensory Characterization of Deep-Fried Fresh-Cut Potatoes Coated with Hydrocolloid/Herbal Extracts

**DOI:** 10.17113/ftb.60.04.22.7691

**Published:** 2022-12

**Authors:** Mia Kurek, Maja Repajić, Mario Ščetar, Lea Radošević, Sandra Pedisić, Zdenka Pelaić, Branka Levaj, Kata Galić

**Affiliations:** Faculty of Food Technology and Biotechnology, University of Zagreb, Pierottijeva 6, 10000 Zagreb, Croatia

**Keywords:** minimally processed potato, edible coatings, nettle leaf extract, olive leaf extract, acrylamide, sensory evaluation

## Abstract

**Research background:**

Recently, natural plant extracts have been used to increase the nutritional value of food and to potentially reduce the absorbed fat and the formation of acrylamide in fried foods. Literature data on the use of edible polymers with nettle or olive leaf extracts are scarce.

**Experimental approach:**

The effect of novel coatings on colour, fat absorption, phenolic and sugar content, and acrylamide formation in deep-fat-fried fresh-cut potatoes was evaluated. Extracts of olive and nettle leaves were incorporated in carboxymethyl cellulose (CMC) and gum arabic, used as coatings for potatoes and applied before frying. This aimed to improve the nutritional quality of deep-fat-fried fresh-cut potatoes.

**Results and conclusions:**

Enrichment of the edible coatings with extracts resulted in a significant change in the visible colour of the potatoes before frying. Significant effect of the extract amount on the sensory characteristics of potatoes was also observed. Most importantly, the perception of characteristic potato odour and taste was not significantly affected by the coating. Although higher amounts of the extract (1.5%) resulted in higher phenolic mass fraction in fried potatoes, the sensory scores decreased. After frying, fat mass fraction in the coated potatoes was reduced by about 15% compared to the uncoated samples. The type of extract affected the total sugar mass fraction in fried potatoes, which was lower in the samples with coatings enriched with olive leaf than in those with nettle leaf. Only gum arabic coating had a reducing effect on acrylamide mass fraction by 17%. Based on all the obtained results, CMC and gum arabic coatings did not influence sensory properties, so they can be recommended as carriers of functional compounds or as a frying pre-treatment for potatoes with favourable effect on fat and acrylamide content.

**Novelty and scientific contribution:**

The knowledge obtained in this study can be exploited for preparation of coatings with functional compounds used as a pre-treatment for fried food with favourable effect on fat and acrylamide content.

## INTRODUCTION

The awareness of a healthy diet with less fat and more antioxidants has become a daily routine for customers. Fried potato products are one of the most commonly consumed foods in everyday life because they are tasty, affordable and easily accessible. Deep-fat frying is a widely and frequently used method for food preparation (*1*, *2*). It results in heat and mass transfer mechanisms that lead to surface browning, rapid water evaporation and oil absorption or degradation (*3*). However, due to the high fat content and the formation of potentially carcinogenic substances, such as acrylamide, fried foods are often considered part of an unhealthy diet (*4*). It has even been recommended by the European Commission that the mass fractions of acrylamide should be below 500 mg/kg in potato strips compared to the guideline benchmark value (*5*). Food scientists are making great efforts to find new technologies to reduce the uptake of unhealthy fat and the formation of acrylamide in starchy fried foods. Since fried foods are an issue of great social relevance, several review articles on current strategies to reduce fat content have been published in recent years (*6*, *7*). Acrylamide is usually formed in carbohydrate-rich foods processed at higher temperatures (above 130 °C) (*8*). Moreover, potatoes are rich in asparagine and reducing sugars, which also affect the formation of acrylamide through Maillard reactions (*9*).

Recent studies on reducing the oil and acrylamide content have revealed three main strategies, which can be grouped as follows: (*i*) changing the frying conditions, *e.g.* lowering the frying temperature (*10*) or increasing the content of antioxidants in the frying oil (*11*), (*ii*) modifying the food surface (*6*, *12*-*14*), *e.g.* edible coatings, and (*iii*) different frying or pre-frying techniques (*15*), such as application of non-thermal technologies (*16*), air frying (*17*), microwave-assisted frying (*18*) or ultrasound treatment (*19*). Edible coatings are characterized by the formation of protective layers on the sample surface, since oil absorption occurs mainly in the crust (surface) during the initial stages of frying. For this purpose, different hydrocolloids have been used (*6*), including pectin (*20*), cellulose-based coatings (*21*), gums (*22*), *etc*. More specifically, edible coatings could serve as carriers for food additives such as antioxidants and antimicrobials on the food surface. To improve the properties of edible coatings, synthetic [butylated hydroxytoluene (BHT) and butylated hydroxyanisole (BHA)] or natural antioxidants can be added. Recently, natural antioxidants have been used to increase the nutritional value, and in some cases, as a tool for reducing acrylamide content in foods (*6*). Natural antioxidants are usually extracted from different plants. Having different polyphenol profiles, the effect on processed foods can be different, such as the formation or inhibition of acrylamide. If acrylamide is eliminated, then the accumulation of carbonyls from the Maillard reaction is reduced (*23*). For example, the combination of gum arabic with turmeric, black pepper, cumin, coriander and red chilli was found to inhibit the formation of acrylamide up to 56% (*24*). Some authors (*12*, *25*) also reported that water-soluble vitamins play an important role in inhibiting the formation of acrylamide. The extract of *Caralluma fimbriata*, an edible succulent cactus, has been shown to stop lipid oxidation by neutralizing free radicals and quenching singlet and triplet oxygen. Thus, the formation of acrylamide during frying at up to 190 °C was reduced (*8*). However, natural essential oils and plant extracts contain volatile chemical compounds with often intense flavour and remarkable colour. Since odour and taste attributes are important factors that influence consumption and overall acceptance of fried foods, this cannot be neglected in the development of novel products, unless they are used as flavour enhancers.

Recently, olive (*Olea europaea* L.) leaves have become the focus of scientific interest to produce new health-promoting functional foods or ingredients (*26*). This by-product of the olive oil industry is rich in polyphenols that can be recovered by extraction. Olive leaf extract is rich in polyphenols that belong specifically to the secoiridoids group (exclusive to the *Oleaceae* family) (*27*).

Stinging nettle (*Urtica dioica* L.) is a plant rich in antioxidants and is very well known as a home remedy in the Mediterranean countries, used as anticancerogenic treatment (*28*) or for food preparations (bread, pasta, *etc*.) (*29*, *30*). Scientific data on its use in edible coatings are very scarce.

The main objective of this study is to examine the efficacy of edible coatings based on two hydrocolloids (carboxymethyl cellulose and gum arabic) and those enriched with nettle and olive leaf extracts on the amount of absorbed fat and formation of acrylamide. Colour, sensory properties and phenolic and sugar profiles of fried potatoes are evaluated.

## MATERIALS AND METHODS

### Materials

Potatoes (*Solanum tuberosum* L. cv. Lady Claire) were supplied by the snack industry (Intersnack Adria Ltd., Hercegovac, Croatia). Cv. Lady Claire is a well-known Dutch industrial potato commonly used in the snack industry. The potatoes were grown and harvested in 2019 in the Slavonia region (Croatia, 45°40'N, 17°1'E). Harvested potatoes were treated with an antisprouting agent (Gro Stop Basis and Gro Stop Fog, Certis Europe B.V., Great Abington, UK) and stored in the dark at 8 °C and relative humidity (RH) of about 100%. Before processing, the potatoes were stored at 16 °C for 3 days. Carboxymethyl cellulose (CMC; V-CMC, Enologica Vason SpA, San Pietro in Cariano, Italy) and gum arabic (Araban® Spray Dry, Enologica Vason) were used as polymers for coatings. Olive (*Olea europea* L.) leaves were collected in the southern Croatian region, while stinging nettle (*Urtica dioica* L.) leaves were collected in the western part of Croatia. The leaves of both plants were used for the preparation of the antioxidant extracts. Sunflower oil (Zvijezda Ltd., Zagreb, Croatia) was used for the frying experiments. Petrol ether (Carlo Erba Reagents S.A.S., LeVaudreil, France) was used for the Soxhlet extractions. Water was of Milli-Q quality (Millipore Corp., Bedford, MA, USA). All organic solvents were of analytical HPLC quality and were purchased from Sigma-Aldrich, Merck (Steinheim, Germany). Chlorogenic acid, catechin, epicatechin, d-(−)-fructose (≥99% GC), d-(+)-glucose (≥99.5% GC), d-(+)-sucrose (≥99.5% GC), acrylamide (>99%) and acrylamide-d3 were also purchased from Sigma-Aldrich, Merck. The chemicals were not additionally purified and only freshly prepared solutions were used.

### Olive and nettle leaf extracts

Olive and nettle leaves were air dried at room temperature and then separately ground using a commercial grinder (GT11; Tefal, Rumilly, France). Extraction was performed using a 14 mm ultrasonic probe (UP200Ht; Hielscher-Ultrasound Technology, Teltow, Germany) for 10 min at 200 W and 100% amplitude with distilled water as extraction solvent. The mass/volume ratio was 1:10. Freshly prepared extracts of olive and stinging nettle leaves were frozen and freeze-dried for 72 h (Alpha 1-4 LSCplus; Martin Christ Gefriertrocknungsanlagen GmbH, Osterode am Harz, Germany). The freeze-dried powder was vacuum packed and stored in the dark until use.

### Preparation of potato samples and coating solutions

Potatoes were peeled by hand, washed and cut into uniform strips of about 10 mm×10 mm×30 mm size using a manual slicer. No chemical washing procedure was applied before and after cutting the potatoes. The strips were then randomly divided into ten groups for coating.

CMC and gum arabic solutions were prepared by dissolving 1 g of polymer powder in distilled water to obtain 1% (*m*/*V*) solutions. The solutions were stirred for 30 min at room temperature ((23±2) °C) until complete dispersion was obtained. For the antioxidant formulations, freeze-dried olive and nettle leaf powders were added to the coating solutions at 0.75 or 1.5% (*m*/*V*) and stirred for 30 min at room temperature to ensure complete solubilisation. Freshly prepared solutions were used for coating.

### Application of the coating solutions and frying

Ten different coating formulations were prepared and used to dip the potatoes (CMC, gum arabic and their mixtures with 0.75 or 1.5% of olive or nettle leaf powder). Potatoes dipped only in distilled water were considered as control group. The samples were soaked for 10 min, then dried at room temperature ((23±2) °C) and RH=55% for 10 min, and finally fried. The whole experiment was done in duplicate.

Coated and uncoated potato strips were deep fried in sunflower oil at (180±2) °C for 10 min in an electric deep-fat fryer (F21-RCS1; Tefal). In each batch, the samples were individually immersed in the frying oil and the oil was changed for each batch. The oil was drained by shaking the frying basket, the samples were allowed to drain on paper towels at room temperature ((23±2) °C) for 2 min and collected for further analysis: colour, water content, fat content, sugar, phenolics and acrylamide content.

### Colour measurement

Colour was measured with a colorimeter (chroma meter CR-5; Konica Minolta, Tokyo, Japan) using the CIELab colour scale: L* (lightness), a* (redness) and b* (yellowness). Eight measurements were taken at 25 °C of each fresh and fried potato strip, and the mean value was recorded. The colour of the control strips was used as a reference. The total colour difference (ΔE*) was calculated as follows:

ΔE*=((Δ*L*)^2^+(Δ*a*)^2^+(Δ*b*)^2^) /1/

where all parameters (ΔL^*2^, Δa*^2^ and Δb*^2^) were calculated with respect to the samples with coating without the extract.

### Sensory analysis

The sensory profile of the raw and fried potato samples was determined by nine panellists following previously given procedure (*31*). Panellists were permanent staff and PhD students (8 women and 2 men, 20-60 years old) previously internally trained for performing sensory analysis on potatoes. Samples were served on coded plastic plates and scored using quantitative descriptive analysis using a discontinuous scale ranging from 0 (no sensation) to 5 (extremely intense). The evaluated parameters of raw potatoes were colour (browning intensity), odour (odour 1=specific potato odour and odour 2=not so specific for potato, but attributed to either the plant or the coating polymer), and texture (firmness and stickiness), while of fried potatoes were colour (browning intensity), odour (odour 1 and odour 2), texture (oiliness, firmness and crispiness) and taste (taste 1=specific potato taste and taste 2=not so specific for potato, but attributed to either the plant or the coating polymer, sweetness, sourness, saltiness and bitterness).

### Determination of moisture and fat content

The fried coated potato strips were ground using a household blender (BKK 2262; Beko, Istanbul, Turkey) and the obtained puree was used to determine the dry matter by drying in an oven at (105±2) °C to constant mass (*32*). Fat content was measured by Soxhlet extraction in petrol ether (*33*). All analyses were performed in duplicate.

### Determination of phenolics

For the ultra performance liquid chromatography (UPLC) analyses, the samples were freeze-dried (Alpha 1-4 LSCPlus; Martin Christ Gefriertrocknungsanlagen GmbH) for 24 h and ground (Waring WSG30; Sprzęt Laboratoryjny i Medyczny Labpartner KBS, Warszawa, Poland). The extraction of phenols was carried out (*34*): the freeze-dried sample (0.5 g) was sonicated with mixed solvent containing 80% methanol and 1% formic acid (5 mL) in an ultrasonic bath (Elmasonic Easy 40H; Elma Schmidbauer GmbH, Singen, Germany) at 50 °C for 30 min. Subsequently, the obtained extract was centrifuged at 1106.82×*g* (Rotofix 32a; Hettich®, Tuttlingen, Germany) for 10 min. The residue was extracted one more time with 5 mL of extraction solvent and the resulting supernatants were combined, filtered into a 10-mL flask and filled up to the mark with the solvent. The obtained extract was filtered using 0.45-µm filter (Macherey-Nagel GmbH & Co. KG, Düren, Germany) before the UPLC MS^2^ analysis.

UPLC analysis was performed using an Agilent 1290 RRLC instrument (Agilent Technologies, Santa Clara, CA, USA) coupled to a binary gradient pump, autosampler and column compartment. The solvent composition and gradient conditions for the determination of phenols were determined previously (*35*). Phenols were separated on a Zorbax Eclipse Plus C18 column (100 mm×2.1 mm, 1.8 µm; Agilent Technologies). Ionization was done by electrospray (ESI) in positive and negative mode (*m/z*=100 to 1000) and the mass spectrometer (QQQ 6430; Agilent Technologies) operated in dynamic multiple reaction monitoring mode (dMRM) with the following source parameters: capillary voltage +4000/-3500 V, nitrogen drying gas temperature at 300 °C with a flow rate of 11 L/h and the nebulizer pressure at 275.790 kPa. Phenols were identified by comparing retention times and mass spectra with those of commercial standards (catechin, epicatechin and chlorogenic acid). External standard calibration was used for quantification. The individual phenols were expressed in mg per 100 g of dry mass. Results are given only for catechin, epicatechin and chlorogenic acid, since they were found as the main compounds in a previous study (*34*). All analyses were performed in duplicate.

### Determination of sugars

Sugars were determined as previously described (*34*). Sugars were extracted by vortexing the freeze-dried sample (0.4 g) and 80% methanol (4 mL). The resulting mixture was then heated in a water bath at 60 °C for 60 min (with occasional vortexing) and centrifuged at 4427.28×*g* for 15 min. The supernatant was filtered into a 5-mL flask and filled up to the mark with the solvent.

Sugars were determined by HPLC on an Agilent 1260 Infinity quaternary LC system (Agilent Technologies, Basel, Switzerland) equipped with a refractive index detector and an automatic injector. An isocratic method with a flow rate of 1 mL/min, a column temperature of 45 °C and 80% acetonitrile (*V*/*V*) as the mobile phase was used to separate sugars on Cosmosil Sugar-D 4.6 IDX250 mm column (Nacalai Tesque, Inc., Kyoto, Japan). Before injection, the extracts were filtered through 0.45-µm membrane filter (Macherey-Nagel GmbH & Co. KG).

The sugars were identified by comparing their retention times with commercial standards (glucose, fructose and sucrose), and quantified by the calibration method with external standards. The results of individual sugars on dry mass basis were expressed in g/100 g. All analyses were performed in duplicate.

### Determination of acrylamide

For the extraction and chromatographic procedure, method by Dite Hunjek *et al.* (*34*) was applied. A mixture of internal acrylamide-d3 standard solution (400 µL, *γ*=1000 ng/mL), water (40 mL) and freeze-dried sample (2.0 g) was manually shaken for 15-30 s, vortexed for 15 s and then stirred for 60 min. The extract was then cooled at 4 °C for 10 min and centrifuged at 1593.82×*g* for 20 min. The supernatant (10 mL) was purified by solid phase extraction (SPE) clean-up procedures using Isolute® Multimode (1 g, 6 mL) and Isolute® ENV+ (0.5 g, 6 mL) SPE columns (International Sorbent Technology, Hengoed, Mid Glamorgan, UK). The obtained extract was then evaporated at 30 °C (Eppendorf™ Concentrator Plus, Fisher Scientific, Leicester, UK) to a volume of approx. 500 µL, filtered through 0.45-µm membrane filter and analysed by UPLC MS^2^.

The acrylamide was determined using an Agilent UPLC system (Agilent 1290 RRLC; Agilent Technologies, Santa Clara) equipped with an electrospray ion source in positive ion mode. The column was a Hypercarb TM (50 mm×2.1 mm, 5 µm) with a guard column (10 mm×2 mm, 5 µm) (Thermo Hypersil-Keystone LLC, Bellefonte, PA, USA). Identification was performed by comparing the peak ratios of multiple reaction monitoring (MRM) transitions at *m*/*z*=54/55 and 44/55 of the sample extracts with standard solutions, and quantification was done using calibration of the internal acrylamide-d3 and acrylamide standards. The results on dray mass basis were expressed in μg/kg. All analyses were performed in duplicate.

### Statistical analysis

Statistical analysis was performed using Statistica v. 12.0 software (*36*). Normality of data was tested using Shapiro-Wilk’s test, and Levene’s test was applied for homoscedasticity test. When appropriate, data were analysed using the ANOVA (parametric data) or Kruskal-Wallis test (nonparametric data). Mean values within groups were compared using Tukey’s HSD test or Kruskal-Wallis test. Spearman’s rank correlation coefficients were calculated to examine the relationships between dependent variables. The significance level for all tests was p≤0.05. Results of statistical analysis are presented as least square (LS) mean value±standard error (S.E.).

## RESULTS AND DISCUSSION

### Colour characteristics of potatoes before and after deep-fat frying

The results of colour properties of fresh-cut potatoes before and after deep-fat frying are given in Table 1. The most significant Spearman’s correlation data, used for understanding deeper results, are not shown in tables.

**Table 1:** Colour parameters of fresh-cut potato before and after frying

Treatment	L*	a*	b*	ΔE*	Browning intensity	L*	a*	b*	ΔE*	Browning intensity
Before					After				
Uncoated	68.2±1.5	-2.1±0.3	18.9±1.2	nd	0.3±0.6	74.7±1.4	-3.6±0.3	20.8±1.6	nd	0.9±1.0
Coated/Source of variation										
Polymer	p=0.208	p=0.177	p=0.505	p=0.06	p=0.666	p=0.241	p=0.247	p=0.038*	p=0.019*	p=0.547
CMC	(62.2±0.6)^a^	(-1.1±0.1)^a^	(16.4±0.4)^a^	(5.3±0.4)^a^	(2.3±0.2)^a^	(68.9±0.6)^a^	(-1.9±0.2)^a^	(23.4±0.4)^b^	(7.6±0.4)^b^	(2.3±0.2)^a^
GA	(63.2±0.4)^a^	(-1.2±0.1)^a^	(17.1±0.4)^a^	(4.4±0.3)^a^	(2.2±0.2)^a^	(69.8±0.6)^a^	(-2.2±0.2)^a^	(22.5±0.3)^a^	(6.3±0.5)^a^	(2.5±0.2)^a^
Extract	p<0.001*	p<0.001*	p=0.002*	p=0.375	p=0.043*	p=0.022*	p=0.048*	p=0.683	p=0.024*	p=0.059
OLE	(63.6±0.4)^b^	(-0.9±0.1)^b^	(17.6±0.4)^b^	(4.6±0.3)^a^	(1.9±0.2)^a^	(70.3±0.5)^b^	(-2.3±0.1)^a^	(23.0±0.4)^a^	(6.2±0.4)^a^	(2.2±0.2)^a^
NLE	(61.7±0.5)^a^	(-1.4±0.1)^a^	(15.9±0.3)^a^	(5.2±0.4)^a^	(2.6±0.2)^b^	(68.4±0.6)^a^	(-1.8±0.2)^b^	(22.9±0.4)^a^	(7.7±0.5)^b^	(2.6±0.2)^a^
Extract content (*m*/*V*)	p<0.001*	p<0.001*	p=0.023*	p<0.001*	p<0.001*	p<0.001*	p<0.001*	p<0.001*	p<0.001*	p<0.001*
0	(64.7±0.6)^c^	(-1.4±0.1)^a^	(16.1±0.5)^a^	(3.3±0.5)^a^	(0.4±0.1)^a^	(73.7±0.3)^b^	(-3.4±0.1)^a^	(24.0±0.5)^b^	(3.8±0.5)^a^	(1.4±0.2)^a^
0.75	(62.9±0.4)^b^	(-0.8±0.1)^b^	(18.1±0.5)^b^	(5.2±0.3)^b^	(2.8±0.2)^b^	(66.9±0.6)^a^	(-2.0±0.1)^b^	(21.1±0.4)^a^	(8.5±0.5)^b^	(2.6±0.2)^b^
1.5	(60.4±0.5)^a^	(-1.1±0.1)^b^	(16.1±0.4)^a^	(6.1±0.4)^b^	(3.6±0.2)^b^	(67.5±0.4)^a^	(-0.8±0.1)^c^	(23.6±0.3)^b^	(8.5±0.4)^b^	(3.3±0.2)^b^
Polymer × extract										
	p=0.062	p=0.003*	p=0.110	p=0.364	p=0.069	p=0.496	p<0.001*	p=0.169	p=0.240	p=0.221
CMC× OLE	(63.1±0.7)^a^	(-0.8±0.1)^b^	(16.9±0.6)^a^	(4.8±0.5)^a^	(1.9±0.2)^a^	(69.4±0.8)^a^	(-2.2±0.2)^a^	(22.9±0.7)^a^	(7.1±0.6)^a^	(2.1±0.2)^a^
CMC × NLE	(61.4±0.9) ^a^	(-1.23±0.1)^a^	(15.8±0.5)^a^	(5.9±0.7)^a^	(2.8±0.4)^a^	(68.5±0.8)^a^	(-1.6±0.3)^b^	(23.8±0.5)^a^	(8.0±0.7)^a^	(2.6±0.3)^a^
	p=0.002*	p<0.001*	p=0.007*	p=0.688	p=0.314	p=0.020*	p=0.201	p=0.030*	p=0.006*	p=0.144
GA × OLE	(64.2±0.4)^b^	(-0.9±0.1) ^b^	(18.2±0.6)^b^	(4.4±0.4)^a^	(1.9±0.3)^a^	(71.3±0.6)^b^	(-2.4±0.2)^a^	(23.1±0.4)^a b^	(5.3±0.6)^a^	(2.2±0.2)^a^
GA × NLE	(62.1±0.5)^a^	(-1.4±0.1)^a^	(16.1±0.4)^a^	(4.5±0.4)^a^	(2.4±0.4)^a^	(68.3±0.9)^a^	(-1.9±0.3)^a^	(21.9±0.5)^a^	(7.3±0.8)^b^	(2.7±0.3)^a^
Polymer × extract content (*m*/*V*)										
	p<0.001*	p<0.001*	p=0.139	p=0.003*	p<0.001*	p<0.001*	p<0.001*	p<0.001*	p<0.001*	p<0.001*
CMC × 0	(65.0±1.1)^b^	(-1.5±0.1)^a^	(16.7±0.8)^a^	(4.1±0.9)^a^	(0.4±0.2)^a^	(73.6±0.3)^b^	(-3.4±0.1)^a^	(25.1±0.8)^b^	(4.6±0.7)^a^	(1.1±0.2)^a^
CMC × 0.75	(62.4±0.5)^b^	(-0.8±0.1)^b^	(17.3±0.6)^a^	(4.8±0.5)^a b^	(2.9±0.2)^b^	(65.9±0.5)^a^	(-1.8±0.1)^b^	(20.8±0.5)^a^	(9.3±0.4)^b^	(2.6±0.2)^b^
CMC × 1.5	(59.3±0.7)^a^	(-0.9±0.1)^b^	(15.1±0.6)^a^	(7.1±0.7)^b^	(3.6±0.3)^b^	(67.3±0.6)^a^	(-0.6±0.2)^c^	(24.2±0.3)^b^	(8.8±0.5)^b^	(3.3±0.3)^b^
	p=0.005*	p=0.002*	p=0.003*	p<0.001*	p<0.001*	p<0.001*	p<0.001*	p=0.025*	p<0.001*	p=0.001*
GA × 0	(64.4±0.5)^b^	(-1.4±0.1)^a^	(15.5±0.4)^a^	(2.5±0.6)^a^	(0.3±0.2)^a^	(73.9±0.5)^b^	(-3.4±0.1)^a^	(23.1±0.5)^b^	(3.0±0.5)^a^	(1.7±0.3)^a^
GA × 0.75	(63.5±0.6)^a b^	(-0.8±0.1)^b^	(18.9±0.8)^b^	(5.6±0.6)^b^	(2.6±0.2)^b^	(67.9±1.1)^a^	(-2.1±0.1)^b^	(21.4±0.6)^a^	(7.6±0.9)^b^	(2.5±0.3)^a b^
GA × 1.5	(61.6±0.5)^a^	(-1.2±0.1)^a^	(17.1±0.4)^a b^	(5.2±0.6)^b^	(3.6±0.3)^b^	(67.6±0.6)^a^	(-1.0±0.2)^c^	(23.0±0.5)^a b^	(8.2±0.5)^b^	(3.3±0.3)^b^

CMC=carboxymethyl cellulose (1%), GA=gum arabic (1%), OLE=olive leaf extract, NLE=nettle leaf extract, nd=not determined. Browning intensity was evaluated by sensory panel. *p≤0.05. The results for the uncoated sample are expressed as mean value±S.D. and the results for coated samples are expressed as mean value±S.E. Values with different letters in superscript within column are statistically different at p≤0.05

The uncoated raw fresh-cut sample had all the colour parameters characteristic of raw potatoes (*31*, *37*). The polymer type had no significant effect on the colour properties of coated samples before frying (Table 1), while the addition of both extract types resulted in significant differences in L*, a* and b*. Both extracts at both applied amounts caused a significant decrease in brightness. The parameter L* also showed a very strong correlation with sensorially assessed browning intensity (Spearman’s rank correlation coefficient r_s_=-0.80). Thus L* could be considered as the best indicator of the browning of fresh-cut potato strips. It could be also taken as the most important quality defect that limits the shelf-life and acceptability of fresh-cut potatoes (*38*). When considering a* and b* values, the addition of both extracts resulted in an increase in a* (although negative values remained), while b* slightly decreased. ΔE* is defined as the overall colour difference with respect to the uncoated sample. Enrichment with both extracts resulted in a significant colour change visible to the human eye (ΔE*>3). Moreover, the overall colour difference was more visible with increasing amount of the extracts. Other authors also showed that L* values were significantly reduced after the treatment of potatoes with rosemary (*39*) or with a green tea essential oil extract (*40*) compared to control samples (soaked in water). The authors also found that increasing the amount of rosemary essential oil above 4% (*m*/*V*) significantly diminished the browning of potatoes. In this study, the results of colour parameters obtained by instrumental analysis were in good agreement with those evaluated by sensory panellists (browning intensity).

After frying, the uncoated samples, which served as control for further comparisons, had a nice goldish colour. Samples with simple coatings and those with both types of extract had lower L* and higher a* values. There was also a very strong correlation between browning intensity and L* (r_s_=-0.66) or a* (r_s_=0.93). Other authors also documented a lower L* value of samples treated with essential oil than of untreated fried potatoes (*39*). Browning intensity increased noticeably in the coated samples after frying compared to the fried uncoated ones (Table 1). This was especially pronounced in the samples coated with gum arabic; however, it was still perceived as a desirable colour of the deep-fat fried potato according to the panellists. Although there was no statistical difference between the extract types, nettle leaf extract had a higher influence on browning and the extract amount showed the most significant (p<0.001) influence on all colour attributes.

### Sensory characteristics of potatoes before and after deep-fat frying

The most important factors to satisfy consumer demand for fried products are sensory quality and nutrient content. According to panellists, the odour and firmness of the raw fresh-cut potato were rated as characteristic for potatoes (Table 2 and Table 3). Stickiness was also rated. Polymer and extract type did not have a significant effect on the firmness and stickiness of the coated samples before frying, while the extract significantly affected stickiness which increased with increasing amount of extract (Table 2). Meanwhile, the firmness of all samples was similar, regardless of the presence of coating (both before and after frying).

**Table 2:** Texture, sensory properties, moisture and fat mass fraction of fresh-cut potato before and after frying

	Stickiness	Firmness		Oiliness	Crispiness	*w*(moisture)/%	*w*(fat)/%
Frying	Before	Before	After	After	After	After	After
Uncoated	0.5±1.3	4.9±0.3	1.7±1.1	1.4±1.1	1.0±0.9	48.5±1.6	10.3±0.1
Coated/Source of variation							
Polymer	p=0.215	p=0.786	p=0.760	p=0.510	p=0.867	p=0.908	p=0.690
CMC	(2.2±0.2)^a^	(4.7±0.1)^a^	(2.0±0.1)^a^	(1.6±0.2)^a^	(1.8±0.1)^a^	(54.8±1.1)^a^	(8.7±0.1)^a^
GA	(1.8±0.2)^a^	(4.7±0.1)^a^	(2.0±0.1)^a^	(1.7±0.2)^a^	(1.8±0.2)^a^	(56.3±2.1)^a^	(8.8±0.2)^a^
Extract	p=0.942	p=0.659	p=0.959	p=0.103	p=0.011*	p=0.166	p=0.127
OLE	(1.9±0.2)^a^	(4.7±0.1)^a^	(2.0±0.1)^a^	(1.5±0.2)^a^	(1.5±0.1)^a^	(55.2±2.1)^a^	(8.9±0.2)^a^
NLE	(2.0±0.2)^a^	(4.7±0.1)^a^	(2.0±0.1)^a^	(1.9±0.2)^a^	(2.1±0.2)^b^	(55.9±1.1)^a^	(8.6±0.2)^a^
Extract content (*m*/*V*)	p=0.004*	p=0.703	p=0.258	p=0.009*	p=0.002*	p=0.867	p=0.142
0	(1.3±0.3)^a^	(4.8±0.1)^a^	(2.1±0.1)^a^	(1.2±0.2)^a^	(1.2±0.2)^a^	(56.4±3.2)^a^	(8.5±0.2)^a^
0.75	(2.3±0.2)^b^	(4.6±0.1)^a^	(2.1±0.2)^a^	(1.8±0.2)^ab^	(2.0±0.2)^b^	(55.3±1.4)^a^	(8.99±0.16)^a^
1.5	(2.4±0.2)^b^	(4.8±0.1)^a^	(1.8±0.2)^a^	(2.0±0.2)^b^	(2.1±0.2)^b^	(54.9±1.3)^a^	(8.7±0.3)^a^
Polymer × extract	p=0.625	p=0.848	p=0.745	p=0.535	p=0.016*	p=0.172	p=0.603
CMC × OLE	(2.0±0.3)^a^	(4.8±0.1)^a^	(1.9±0.2)^a^	(1.5±0.2)^a^	(1.4±0.2)^a^	(52.9±1.4)^a^	(8.6±0.2)^a^
CMC × NLE	(2.3±0.3)^a^	(4.7±0.1)^a^	(2.0±0.2)^a^	(1.7±0.2)^a^	(2.2±0.2)^b^	(56.6±1.5)^a^	(8.8±0.2)^a^
	p=0.616	p=0.430	p=0.835	p=0.112	p=0.224	p=1.000	p=0.009*
GA × OLE	(1.9±0.2)^a^	(4.7±0.1)^a^	(2.0±0.2)^a^	(1.5±0.2)^a^	(1.6±0.2)^a^	(57.4±3.9)^a^	(9.2±0.3)^b^
GA × NLE	(1.8±0.3)^a^	(4.7±0.1)^a^	(2.0±0.2)^a^	(2.0±0.2)^a^	(2.0±0.3)^a^	(55.2±1.7)^a^	(8.4±0.2)^a^
Polymer × extract content (*m*/*V*)	p=0.072	p=0.945	p=0.581	p=0.006*	p=0.005*	p=0.513	p=0.734
CMC × 0	(1.4±0.4)^a^	(4.8±0.1)^a^	(2.1±0.1)^a^	(0.9±0.2)^a^	(1.1±0.2)^a^	(52.9±2.0)^a^	(8.6±0.3)^a^
CMC × 0.75	(2.4±0.3)^a^	(4.7±0.2)^a^	(2.0±0.3)^a^	(1.8±0.2)^ab^	(2.1±0.3)^b^	(55.1±2.2)^a^	(8.7±0.1)^a^
CMC × 1.5	(2.7±0.3)^a^	(4.8±0.1)^a^	(1.8±0.2)^a^	(2.2±0.3)^b^	(2.2±0.2)^b^	(56.4±1.7)^a^	(8.8±0.2)^a^
	p=0.059	p=0.380	p=0.395	p=0.458	p=0.216	p=1.000	p=0.056
GA × 0	(1.2±0.3)^a^	(4.9±0.1)^a^	(2.0±0.2)^a^	(1.4±0.3)^a^	(1.3±0.3)^a^	(59.8±5.8)^a^	(8.4±0.3)^a^
GA × 0.75	(2.1±0.3)^a^	(4.6±0.2)^a^	(2.2±0.3)^a^	(1.9±0.3)^a^	(2.0±0.3)^a^	(55.5±2.1)^a^	(9.3±0.2)^a^
GA × 1.5	(2.1±0.3)^a^	(4.7±0.1)^a^	(1.8±0.3)^a^	(1.9±0.3)^a^	(2.0±0.2)^a^	(53.5±1.8)^a^	(8.7±0.5)^a^

CMC=carboxymethyl cellulose (1%), GA=gum arabic (1%), OLE=olive leaf extract, NLE=nettle leaf extract. *p≤0.05. The results for the uncoated sample are expressed as mean value±SD and the results for coated samples are expressed as mean value±SE. Values with different letters within column are statistically different at p≤0.05

**Table 3:** Odour and taste sensory properties of fresh-cut potato before and after frying

	Odour 1	Odour 2	Odour 1	Odour 2	Taste 1	Taste 2	Sweetness		Sourness	Saltiness	Bitterness
Frying	Before		After		After		After			After	
Uncoated	4.6±0.7	0.0±0.0	4.2±0.9	0.2±0.7	4.1±1.3	0.6±1.1	0.8±1.6	0.1±0.3		0.9±1.2	0.7±1.0
Coated/Source of variation											
Polymer	p=0.453	p=0.906	p=0.597	p=0.931	p=0.694	p=0.387	p=0.322	p=0.757		p=0.657	p=0.492
CMC	(3.7±0.27)^a^	(0.8±0.2)^a^	(3.9±0.1)^a^	(0.5±0.1)^a^	(3.7±0.2)^a^	(0.8±0.2)^a^	(0.9±0.2)^a^	(0.4±0.1)^a^		(0.7±0.1)^a^	(0.8±0.1)^a^
GA	(3.5±0.2)^a^	(0.9±0.2)^a^	(3.8±0.1)^a^	(0.5±0.1)^a^	(3.70±0.)^a^	(0.5±0.1)^a^	(1.1±0.2)^a^	(0.4±0.1)^a^		(0.7±0.1)^a^	(0.9±0.2)^a^
Extract	p=0.082	p=0.545	p=0.394	p=0.899	p=0.718	p=0.306	p=0.461	p=0.037*		p=0.326	p=0.906
OLE	(3.8±0.2)^a^	(0.9±0.2)^a^	(3.9±0.1)^a^	(0.6±0.1)^a^	(3.7±0.2)^a^	(0.8±0.2)^a^	(1.0±0.2)^a^	(0.3±0.1)^a^		(0.7±0.1)^a^	(0.9±0.2)^a^
NLE	(3.5±0.2)^a^	(0.±0.1)^a^	(3.8±0.1)^a^	(0.5±0.1)^a^	(3.±0.2)^a^	(0.5±0.1)^a^	(1.1±0.2)^a^	(0.5±0.1)^b^		(0.8±0.1)^a^	(0.8±0.1)^a^
Extract content (*m*/*V*)	p<0.001*	p<0.001*	p<0.001*	p=0.002*	p=0.095	p=0.224	p=0.147	p=0.020*		p=0.648	p=0.199
0	(4.5±0.2)^b^	(0.3±0.2)^a^	(4.3±0.1)^b^	(0.2±0.1)^a^	(3.9±0.2)^a^	(0.8±0.2)^a^	(1.4±0.3)^a^	(0.2±0.1)^a^		(0.9±0.2)^a^	(0.8±0.2)^a^
0.75	(3.4±0.2)^a^	(0.9±0.1)^b^	(3.±0.1)^ab^	(0.4±0.1)^ab^	(3.8±0.2)^a^	(0.4±0.2)^a^	(1.0±0.)^a^	(0.4±0.1)^ab^		(0.8±0.2)^a^	(0.7±0.2)^a^
1.5	(3.±0.2)^a^	(1.3±0.2)^b^	(3.57±0.)^a^	(0.9±0.2)^b^	(3.4±0.2)^a^	(0.8±0.2)^a^	(0.7±0.2)^a^	(0.6±0.1)^b^		(0.6±0.1)^a^	(1.1±0.2)^a^
Polymer × extract	p=0.157	p=0.559	p=0.193	p=0.736	p=0.465	p=0.508	p=0.862	p=0.008*		p=0.227	p=0.977
CMC × OLE	(3.9±0.3)^a^	(0.8±0.2)^a^	(4.1±0.2)^a^	(0.5±0.2)^a^	(3.9±0.2)^a^	(0.9±0.3)^a^	(1.0±0.3)^a^	(0.2±0.1)^a^		(0.6±0.2)^a^	(0.8±0.2)^a^
CMC × NLE	(3.6±0.2)^a^	(0.9±0.2)^a^	(3.8±0.2)^a^	(0.5±0.1)^a^	(3.6±0.3)^a^	(0.6±0.2)^a^	(0.9±0.2)^a^	(0.6±0.1)^b^		(0.8±0.2)^a^	(0.7±0.2)^a^
	p=0.298	p=0.791	p=0.891	p=0.619	p=0.822	p=0.420	p=0.396	p=0.730		p=0.803	p=0.842
GA × OLE	(3.6±0.3)^a^	(0.85±0.23)^a^	(3.83±0.18)^a^	(0.59±0.17)^a^	(3.63±0.23)^a^	(0.67±0.20)^a^	(1.04±0.26)^a^	(0.41±0.14)^a^		(0.74±0.19)^a^	(1.04±0.25)^a^
GA × NLE	(3.4±0.2)^a^	(0.85±0.20)^a^	(3.85±0.16)^a^	(0.44±0.14)^a^	(3.78±0.20)^a^	(0.41±0.14)^a^	(1.22±0.22)^a^	(0.41±0.12)^a^		(0.78±0.19)^a^	(0.91±0.22)^a^
Polymer × extract content (*m*/*V*)	p<0.001*	p=0.009*	p=0.002*	p<0.001*	p=0.239	p=0.718	p=0.322	p=0.156		p=0.638	p=0.357
CMC × 0	(4.7±0.2)^b^	(0.4±0.3)^a^	(4.3±0.2)^b^	(0.0±0.0)^a^	(3.9±0.4)^a^	(0.9±0.3)^a^	(1.2±0.4)^a^	(0.2±0.2)^a^		(1.0±0.3)^a^	(0.7±0.2)^a^
CMC × 0.75	(3.5±0.2)^a^	(0.8±0.2)^ab^	(4.1±0.2)^ab^	(0.6±0.2)^ab^	(3.8±0.2)^a^	(0.6±0.3)^a^	(1.1±0.3)^a^	(0.3±0.1)^a^		(0.6±0.2)^a^	(0.6±0.2)^a^
CMC × 1.5	(3.1±0.3)^a^	(1.3±0.3)^b^	(3.4±0.2)^a^	(1.1±0.2)^b^	(3.5±0.3)^a^	(0.8±0.3)^a^	(0.5±0.2)^a^	(0.5±0.2)^a^		(0.5±0.2)^a^	(1.0±0.3)^a^
	p=0.004*	p=0.004*	p=0.062	p=0.632	p=0.307	p=0.247	p=0.340	p=0.131		p=0.877	p=0.528
GA × 0	(4.3±0.3)^b^	(0.2±0.2)^a^	(4.2±0.2)^a^	(0.4±0.2)^a^	(3.9±0.2)^a^	(0.7±0.2)^a^	(1.6±0.4)^a^	(0.2±0.2)^a^		(0.8±0.2)^a^	(1.0±0.3)^a^
GA × 0.75	(3.3±0.3)^a^	(1.0±0.2)^b^	(3.8±0.2)^a^	(0.4±0.2)^a^	(3.8±0.3)^a^	(0.2±0.1)^a^	(1.0±0.2)^a^	(0.4±0.1)^a^		(0.9±0.3)^a^	(0.8±0.3)^a^
GA × 1.5	(3.0±0.4)^a^	(1.3±0.3)^b^	(3.5±0.2)^a^	(0.7±0.2)^a^	(3.4±0.3)^a^	(0.7±0.3)^a^	(0.8±0.2)^a^	(0.6±0.2)^a^		(0.6±0.2)^a^	(1.1±0.2)^a^

CMC=carboxymethyl cellulose (1%), GA=gum arabic (1%), OLE=olive leaf extract, NLE=nettle leaf extract, nd=not determined. *p≤0.05. Results for the uncoated sample are expressed as mean±S.D. and results for coated samples are expressed as mean±S.E. Values with different letters within column are statistically different at p≤0.05

Although oiliness was less pronounced at lower extract amounts, crispiness was higher in samples coated with higher extract amounts. This was considered as a positive effect when compared to control. Accordingly, a very strong positive correlation was found between crispness and oiliness (r_s_=0.93) as well as between browning intensity and crispiness (r_s_=0.90). Similar to this study, gum arabic was found to increase the crispness of coated potato chips more than CMC (*13*).

As for the firmness and stickiness before frying, polymer and extract type had no significant effect on odour 1 and 2, while extract amount significantly decreased odour 1 and increased odour 2 (Table 3). A very strong negative correlation was calculated between browning intensity and potato odour (r_s_=-0.92). Edible coatings aim to improve the texture of fried food.

After frying, further sensory changes occurred in terms of odour and taste characteristics (Table 3). Samples with extract-enriched coatings had less characteristic potato odour and more intense sourness, especially the samples with added nettle leaf extract. This is due to the fact that the used extracts were mainly composed of polyphenols known for their bitter taste. The perception of potato odour (odour 1) and taste (taste 1) was not significantly affected by the polymer CMC and gum arabic. For the uncoated samples, odour 1 value was 4.22, then it decreased to 3.94 and 3.84 for samples treated with CMC and gum arabic, respectively, and to 3.96 and 3.81 for samples treated with olive leaf and nettle leaf extract, respectively. The opposite behaviour was observed for odour 2. Just like for sour taste, taste 2 was more pronounced in the coated samples, with no significant differences between the coating formulations. A strong correlation was observed between odour 2 and odour 1 (r_s_=-0.85) and odour 1 and taste 1 (r_s_=0.79), indicating that as potato odour decreased, odour 2 was more pronounced. This was even enhanced at the higher extract (1.5%) amount. An effect of rosemary oil on typical potato taste was already shown previously (*39*), but without consistent trend. Sweetness was higher in the coated samples than in the uncoated samples (with higher values for gum arabic), except for CMC with 1.5% olive leaf or nettle leaf extract. Since sweetness showed a strong positive correlation (r_s_=0.74) with taste 1, it can be considered as a favourable characteristic of potato. The coated samples showed a slightly higher saltiness, without significant differences between the samples. Correlation with sensory properties was observed as follows: odour 1 with browning (r_s_=-0.82), odour 2 with browning (r_s_=0.74) and sourness with browning (r_s_=0.72). Even though perceivable changes of some quality parameters were noticeable, the overall organoleptic quality and acceptability of potatoes coated with simple and functionalised (with extract) coatings was good.

### Moisture and fat content in fresh-cut deep-fat-fried potato

Moisture mass fraction of coated fried samples was higher (grand mean value 55.53%, Table 2) than of the uncoated sample (48.49%), while fat mass fraction decreased by 15%. No significant differences in moisture or fat mass fraction were observed between CMC and gum arabic or between extract amounts. The results were consistent with those reported previously (*41*), although they may vary depending on the potato variety, coating type and frying conditions. For example, it was found that guar gum coating reduced fat content in fried potatoes by up to 51.8% (*41*), while in another study an increase in fat content was measured in pectin-coated fried potatoes (56.1%) compared to the control (*42*).

### Phenolics in fresh-cut deep-fat-fried potato

The predominant phenolic compounds that accounted for 80% of the total phenolics in raw potatoes on dry mass basis were chlorogenic acid (12.1 mg/100 g), epicatechin (0.810 mg/100 g) and catechin (0.215 mg/100 g) (Table 4). The major phenolic compounds identified in the uncoated potato were similar to those identified in a previous study of the cultivar Lady Claire (*34*). During frying of the uncoated samples, the mass fractions of catechin, epicatechin and chlorogenic acid changed (Table 4). The most remarkable decrease was observed of chlorogenic acid (by about 50%) and catechin (by about 35%), while the mass fraction of epicatechin slightly increased. During the exposure to high temperatures, it is possible that phenolics are released from degraded cells (*43*).

**Table 4:** Mass fractions (on dry mass basis) of phenolics, sugars and acrylamide in fresh-cut potato before (BF) and after frying (AF)

		*w*(catechin)/ (mg/100 g)		*w*(epicatechin)/ (mg/100 g)	*w*(chlorogenic acid)/(mg/100 g)	*w*(fructose)/ (g/100 g)	*w*(glucose)/ (g/100 g)	*w*(sucrose)/ (g/100 g)	*w*(acrylamide)/(µg/kg)
Uncoated	BF		0.215±0.007	0.810±0.008	12.1±0.0	0.069±0.002	0.170±0.020	0.210±0.020	nd
	AF		0.140±0.006	0.877±0.004	6.6±0.0	0.072±0.003	0.077±0.004	0.155±0.005	116±16
Coated AF/Source of variation									
Polymer		p=0.662		p=0.298	p=0.127	p<0.001*	p=0.298	p=0.488	p=0.419
CMC		(0.220±0.003)^a^		(1.05±0.04)^a^	(7.3±0.6)^a^	(0.160±0.020)^b^	(0.190±0.020)^a^	(0.182±0.008)^a^	(254±25)^a^
GA		(0.217±0.002)^a^		(1.01±0.06)^a^	(8.3±0.4)^a^	(0.080±0.005)^a^	(0.154±0.005)^a^	(0.190±0.010)^a^	(299±50)^a^
Extract		p=0.726		p=0.166	p=0.860	p=0.326	p=0.011*	p<0.001*	p=0.206
OLE		(0.219±0.002)^a^		(1.09±0.06)^a^	(7.8±0.4)^a^	(0.120±0.020)^a^	(0.160±0.020)^a^	(0.164±0.009)^a^	(300±42)^a^
NLE		(0.218±0.002)^a^		(0.97±0.03)^a^	(7.9±0.6)^a^	(0.130±0.020)^a^	(0.180±0.010)^b^	(0.205±0.004)^b^	(253±37)^a^
Extract content (*m*/*V*)		p=0.076		p=0.523	p=0.043*	p=0.914	p=0.047*	p=0.833	p=0.001*
0		(0.215±0.002)^a^		(1.05±0.04)^a^	(7.0±0.1)^a^	(0.170±0.040)^b^	(0.210±0.020)^b^	(0.198±0.001)^a^	(147±20)^a^
0.75		(0.217±0.002)^a^		(1.05±0.09)^a^	(7.4±0.9)^ab^	(0.099±0.006)^a^	(0.160±0.010)^a^	(0.170±0.020)^a^	(283±49)^ab^
1.5		(0.224±0.004)^a^		(1.00±0.05)^a^	(9.0±0.4)^b^	(0.096±0.006)^a^	(0.150±0.008)^a^	(0.180±0.010)^a^	(399±15)^b^
Polymer × extract		p=0.859		p=0.077	p=0.255	p=0.748	p=0.018*	p=0.025*	p=0.183
CMC × OLE		(0.221±0.004)^a^		(1.10±0.05)^a^	(7.1±0.6)^a^	(0.160±0.040)^a^	(0.170±0.030)^a^	(0.160±0.010)^a^	(259±28)^a^
CMC × NLE		(0.220±0.004)^a^		(1.00±0.05)^a^	(7.5±1.0)^a^	(0.160±0.040)^a^	(0.200±0.020)^b^	(0.199±0.004)^b^	(248±43)^a^
		p=0.936		p=1.000	p=1.000	p=0.199	p=0.054	p=0.010*	p=0.520
GA × OLE		(0.217±0.002)^a^		(11.0±0.10)^a^	(8.4±0.5)^a^	(0.071±0.002)^a^	(0.144±0.004)^a^	(0.160±0.020)^a^	(340±79)^a^
GA × NLE		(0.217±0.002)^a^		(0.94±0.02)^a^	(8.3±0.7)^a^	(0.090±0.009)^a^	(0.165±0.008)^a^	(0.211±0.005)^b^	(259±65)^a^
Polymer × extract content (*m*/*V*)		p=0.062		p=0.082	p=0.007*	p=0.023*	p=0.018*	p=0.777	p=0.024*
CMC × 0		(0.214±0.003)^a^		(1.15±0.00)^a^	(7.2±0.0)^ab^	(0.272±0.005)^b^	(0.270±0.020)^b^	(0.198±0.001)^a^	(199±5)^a^
CMC × 0.75		(0.217±0.002)^a^		(0.93±0.01)^a^	(5.2±0.1)^a^	(0.104±0.006)^a^	(0.150±0.010)^a^	(0.180±0.010)^a^	(199±22)^a^
CMC × 1.5		(0.230±0.005)^a^		(1.08±0.08)^a^	(9.5±0.5)^b^	(0.109±0.003)^ab^	(0.160±0.010)^ab^	(0.170±0.020)^a^	(363±13)^b^
		p=0.809		p=0.736	p=0.018*	p=0.020*	p=0.197	p=1.000	p=0.024*
GA × 0		(0.215±0.003)^a^		(0.95±0.00)^a^	(6.8±0.0)^a^	(0.064±0.001)^a^	(0.157±0.005)^a^	(0.199±0.001)^a^	(95±3)^a^
GA × 0.75		(0.218±0.003)^a^		(1.20±0.20)^a^	(9.6±0.5)^b^	(0.090±0.010)^b^	(0.164±0.015)^a^	(0.170±0.030)^a^	(368±78)^b^
GA × 1.5		(0.218±0.003)^a^		(0.91±0.05)^a^	(8.6±0.7)^ab^	(0.084±0.006)^ab^	(0.142±0.003)^a^	(0.190±0.010)^a^	(435±9)^b^

CMC=carboxymethyl cellulose (1%), GA=gum arabic (1%), OLE=olive leaf extract, NLE=nettle leaf extract, nd=not determined. *p≤0.05. Results for the uncoated sample are expressed as mean±S.D. and results for coated samples are expressed as mean±S.E. Values with different letters within column are statistically different at p≤0.05

The mass fraction of total phenolics did not differ significantly in the samples coated with different polymer or extract type. The only significant difference, more specifically an increase, was observed in the mass fraction of chlorogenic acid in the samples treated with a higher extract amount.

After frying, higher mass fraction of phenolics was found in the coated potatoes. This was especially evident for catechin and epicatechin. Higher mass fractions of chlorogenic acid were found only in the coated potatoes than in the uncoated ones after frying. These results could be due to the phenolic content of the extracts or protective role of the coatings. The mechanism of activity of phenolic compounds was previously given in scientific literature, but the specific syntheses and performance of their antioxidant mechanism during frying still remains unclear (*44*). In olive leaves, oleuropein was a predominant compound, followed by rutin, verbascoside, hydroxytyrosol, caffeic acid and chlorogenic acid (*45*). In nettle leaves, cinnamic acids were the most abundant group, followed by flavonols (mainly derivatives of kaempferol and quercetin), flavones, flavan-3-ols (catechin and epicatechin), benzoic acids, coumarins, isoflavones and other acids (*46*). However, chlorogenic acid, catechin and epicatechin were present in olive leaves and nettle, respectively, at amounts too low to affect their mass fraction in potato samples. In contrast, extract amount affected colour parameters and browning. As phenolics are known to be responsible for browning processes, further studies are needed to better understand the mechanism of all these changes.

### Sugars and acrylamide in deep fat fried potato

Main sugars found in potato samples were monosaccharides (glucose and fructose) and disaccharides (sucrose) (Table 4). Their mass fractions were in the following order: sucrose>glucose>fructose. The mass fraction of sucrose and glucose was lower in fried uncoated potatoes than in the raw potatoes, while the mass fraction of fructose remained the same. The decrease in sugar mass fraction during frying was probably due to the involvement of these sugars in Maillard reactions. Here, interactions of sugars with amino acids leads to the formation of brownish compounds and to the characteristic odour and taste of the fried potato (*34*). A significant difference between polymer coatings was only found for fructose mass fraction, which was higher in CMC-coated samples. The extract type seemed to have an influence on glucose and sucrose. The amount of extract significantly affected only the glucose mass fraction, which is an important precursor in the synthesis of acrylamide. In addition, all sugars were present in all coated samples in the same amount as in the uncoated samples before frying. The same question arises as with phenolics: did the coatings and extracts have any protective role on the sugars or was this a consequence of their composition? It is possible that the used extracts contained some minor components that remained during the extraction process and were retained in the lyophilised powder, apart from the polyphenols, which could affect the amount of sugar (*26*) and thus the acrylamide content.

Acrylamide is considered as a potentially carcinogenic compound (*28*). According to the European Commission Regulation 2017/2158 (*5*), the benchmark value for acrylamide in potato products is 500 μg/kg on fresh mass basis. It should be noted that the acrylamide content did not exceed the permitted value in all samples (Table 4). According to the literature, reducing sugars play a crucial role in acrylamide synthesis (*47*). With increasing content of sugars in potato, aside changes in colour and bitterness, this also leads to the formation of acrylamide in fried potato. At temperatures above 120 °C and with decreasing the water content, acrylamide can be formed as a result of interactions of glucose and fructose with asparagine (*48*).

In this study, the mass fraction of reducing sugars was higher in CMC- than in gum arabic-treated potatoes and lower acrylamide values were found in the samples coated with gum arabic (95 μg/kg) than in the uncoated samples (115.89 μg/kg), while CMC was not as effective as gum arabic. It is possible that gum arabic formed more uniform coating on potato surface than CMC that might have modulated heat transfer from the frying oil to potato surface and then the mass fraction of formed acrylamide was reduced. The relation could also be made with the sugar mass fraction that was found to be higher in the samples coated with CMC than with gum arabic. Since the mass fraction of sugars plays an important role in the formation of acrylamide, CMC had less pronounced inhibitory effect than gum arabic. According to literature, soaking in gum arabic (1%, *m*/*V*) for 60 min reduced the content of acrylamide in fried potatoes by 20% (*24*), soaking in alginic acid solution at 1% (*m*/*V*) for 5 h reduced it by 30% and in 5% (*m*/*V*) solution for 1 h by 60% (*20*), while the use of other biopolymer, *i.e.* alginate (0.3%), pectin (0.2%) and chitosan (1%) reduced acrylamide by 54, 51 and 41%, respectively (*14*). The most likely explanation is that coatings with hydrocolloids as the main component alter the texture of foods due to their gelling or thickening properties. Then, they disrupt the molecular interactions between the precursors of acrylamide (*e.g.* glucose and asparagine) with different efficacy (*20*, *24*).

Treatment with coatings containing extracts significantly increased the acrylamide content. Although there are examples in the literature of positive effect of different plant extracts on the reduction of acrylamide, the present study could not confirm these results. The discrepancy between the results could be due to the different chemical composition of the used plants and thus produced extracts (*8*, *49*). Literature data also suggest that the patterns of relative inhibitory activities depend on the type of coating, amount and soaking time (*14*, *50*). Even though some authors (*8*) showed that *C. fimbriata* was effective in decreasing acrylamide content (42.5 μg/kg) in immersed samples, and thus improved nutritional quality, this was not the case in the present study where no positive effect on the nutritional value of fried samples was observed. Literature data (*51*) showed that ginger, borage and fennel reduced acrylamide by 21.91, 66.29 and 29.15%, respectively, while in another study (*52*) it was found that the *Allium hertifolium* extract was more effective than that of *Zataria multiflora* in fried potato crisps. Knowledge on the mechanism of action of olive leaf and nettle leaf extracts remains preliminary for further studies, highlighting the importance of testing novel coating formulations and the variability of results in the scientific literature.

## CONCLUSIONS

From all the tested parameters, it appears that the application of simple coatings did not significantly affect potato colour (instrumentally measured or sensory evaluated), odour and taste. On the contrary, increasing olive and nettle leaf extract amount led to perceivable changes in the organoleptic quality, but it was not so important to affect overall acceptability. It appears that the application of simple coatings can effectively reduce fat absorption in fried fresh-cut potatoes, but most of them did not minimise the formation of acrylamide. Fat mass fraction in the coated potatoes without extracts was reduced by about 15% after frying, while the extracts of olive and nettle leaves incorporated into edible coatings showed no effect on fat content. The type of coating and extract did not significantly affect phenolic mass fraction, but in general it did affect the mass fraction of sugar in the fried potatoes. Thus, samples with gum arabic coatings had lower sugar mass fraction than those with carboxymethyl cellulose (CMC) coatings. Samples enriched with olive leaf extract also had lower sugar mass fraction than those enriched with nettle leaf extract. Using olive or nettle extract has the potential to improve the biological quality of deep-fat-fried fresh-cut potatoes because higher amount of the extract results in higher phenolic mass fraction in fried potatoes, while further investigation is needed to create a formulation with a generally beneficial effect. Only the simple gum arabic coating showed a reducing effect on acrylamide mass fraction by 17%, showing also lower sugar mass fraction. Although we expected the effect on fat reduction to be higher, the obtained results can serve as a good indication for further research. Based on all obtained results, CMC and even simpler gum arabic coating can be recommended as carriers of functional compounds or as a pre-treatment of fried potatoes with favourable effect on fat and acrylamide content without affecting sensorial properties.
